# Increase in the flock prevalence of lameness in ewes is associated with a reduction in farmers using evidence-based management of prompt treatment: A longitudinal observational study of 154 English sheep flocks 2013–2015

**DOI:** 10.1016/j.prevetmed.2019.104801

**Published:** 2019-12-01

**Authors:** Naomi S. Prosser, Kevin J. Purdy, Laura E. Green

**Affiliations:** a School of Life Sciences, Gibbet Hill Campus, The University of Warwick, Coventry, CV4 7AL, United Kingdom; b College of Life and Environmental Sciences, University of Birmingham, Edgbaston, Birmingham, B15 2TT, United Kingdom

**Keywords:** Lameness, Prevalence, Population attributable fraction, Risk factors, Sheep, Treatment

## Abstract

Since 2006, farmers in England have received new recommendations on best practice to manage lameness in sheep through a range of knowledge exchange activities. The adoption of each recommendation varied, but in 2013 approximately 50% of farmers reported treating all lame sheep within 3 days of onset of lameness (prompt treatment), 41% did not practice routine foot trimming, 50% culled sheep that had been lame and 14% vaccinated against footrot; all recommended best practices. The aim of this study was to investigate the prevalence of lameness in ewes in England from 2013 to 2015 and to identify changes in practice to manage lameness between 2013 and 2015 and the population attributable fraction for these managements.

A longitudinal study with a cohort of 154 English sheep farmers was run for three years, farmers completed questionnaires on lameness in their flock for the previous 12 months in 2013, 2014 and 2015. The geometric mean prevalence of lameness in ewes was 4.1% in 2015, significantly higher than 3.3% and 3.2% for the same 128 farmers who provided data in both 2013 and 2014. Between 2013 and 2015 there was a significant reduction in farmers practising prompt treatment (50.6%–28.6%) but an increase in not practising routine foot trimming (40.9%–79.2%), culling sheep that had been lame (49.4%–81.8%), and vaccinating against footrot (14.3%–29.2%).

Not practising prompt treatment, ≥5% of sheep feet bleeding during routine foot trimming, vaccinating ewes for <6 years or not vaccinating at all, and other flocks mixing with the flock, were associated with a significantly higher flock prevalence of lameness. Culling sheep that had been lame was not associated with prevalence of lameness. The population attributable fractions (PAFs) for not vaccinating for>5 years, not treating lame sheep promptly, ≥5% of sheep feet bleeding during routine foot trimming, and mixing of flocks were 34.5%, 25.3%, 2.9% and 2.4%. In 2013, when 50% of farmers used prompt treatment, the PAF for not using prompt treatment was only 13.3%. We conclude that the change in practice by these farmers towards flock-level managements and a reduction in individual prompt treatment of lame sheep negatively impacted the prevalence of lameness in sheep. This change occurred despite the evidence that prompt treatment of lame sheep is highly effective at reducing the prevalence of lameness in sheep flocks and is an example of cognitive dissonance.

## 1 Introduction

In the UK, lameness in sheep costs the sheep industry £80 – £85 million per annum (Wassink et al., 2010b; Winter and Green, 2017). Economic losses arise from both treatment costs and reduced production (Wassink et al., 2010b; Winter and Green, 2017). In the UK, footrot, both interdigital dermatitis (ID) and severe footrot (SFR), caused by *Dichelobacter nodosus* (Beveridge, 1941) is present in > 90% of flocks and causes approximately 70% of lameness (Winter et al., 2015). Contagious ovine digital dermatitis (CODD) is another infectious cause of lameness that is present in 35%–60% of flocks (Angell et al., 2014; Dickins et al., 2016). CODD accounts for approximately 30% of lameness in affected flocks (Dickins et al., 2016). There are other non-infectious causes of lameness such as granulomas and foot abscesses which cause < 1% of lameness (Winter et al., 2015).

Lameness in sheep is a welfare concern because lame sheep are in pain (Ley et al., 1989). Recognising the welfare implications of lameness and the recent advances in best practice treatments and managements, the Farm Animal Welfare Council (FAWC) set targets for the flock prevalence of lameness in sheep in the UK to be ≤ 5% by 2016 and ≤ 2% by 2021 (FAWC, 2011). This target was based on evidence that the prevalence of lameness can be reduced to < 2% (Wassink et al., 2010b) in flocks using prompt (within 3 days of onset of lameness) and appropriate (topical and parenteral antimicrobial) treatment of lame sheep (Kaler et al., 2010) without therapeutic or routine foot trimming, footbathing or vaccination (summarised in Green and Clifton, 2018).

The global mean prevalence of lameness in sheep was 10.2% in 2004 (Kaler and Green, 2009). This had fallen to 4.9% in 2013 as a greater proportion of farmers used the evidence-based managements to treat and control lameness (Winter et al., 2015). Despite 50% of farmers practising prompt treatment, i.e. treating all lame sheep within 3 days of onset of lameness, this management had the highest population attributable fraction (PAF) for lameness of 13.3% in 2013 (Grant et al., 2018). Farmers report that prompt treatment is difficult because of the need to catch the sheep to treat them (O’Kane et al., 2017; Grant et al., 2018), and in 2007, 161 farmer respondents reported that they would prefer to avoid individual treatments and use the whole flock managements of footbathing and vaccination. However, these same farmers were unsatisfied with the efficacy of footbathing and vaccination and satisfied with individual treatments (Wassink et al., 2010a); this is an example of cognitive dissonance. A discussion point in the Wassink et al. (2010a) study was that those involved in knowledge exchange on lameness would need to be aware that farmers would more readily adopt whole flock practices that they preferred rather than evidence-based practices of individual treatment, and so it would be important to present the evidence clearly and explain the need for prompt treatment of individual lame sheep.

One flock management that the 161 farmers said they would prefer to reduce was routine foot trimming (Wassink et al., 2010a) which is associated with a higher prevalence of lameness because over trimming causes feet to bleed (Winter et al., 2015; Dickins et al., 2016; Grant et al., 2018). Indeed, subsequently, in a one-year intervention study of 884 farmers from 2013 to 2014, the greatest change in behaviour to manage lameness was a significant reduction in the proportion of farmers practising routine foot trimming (Grant et al., 2018). In the same study, there was also a small increase in the proportion of farmers who caught lame sheep promptly (Grant et al., 2018).

Other managements associated with a lower prevalence of lameness include vaccinating against footrot and quarantining new sheep for > 3 weeks (Winter et al., 2015). Culling sheep that had been lame was not associated with the prevalence of lameness in ewes in 2013 (Winter et al., 2015), but in another study, culling ewes at the start of a control programme was associated with a reduction in lameness (Witt and Green, 2018).

The FAWC target of a global mean flock prevalence of lameness of ≤ 5% by 2016 was achieved in 2013 (Winter et al., 2015), however, further uptake of best practices is still needed to reduce the flock prevalence of lameness to ≤ 2%. The aims of the current study were to investigate the prevalence of lameness and change in management of lameness in sheep flocks monitored from 2013 to 2015, and to estimate the population attributable fractions that would inform on key management practices that could reduce the national global flock prevalence of lameness to ≤ 2% by 2021 if more widely adopted.

## 2 Material and methods

Ethical approval was obtained from The University of Warwick Biomedical & Scientific Research Ethics Committee (reference number: REGO-2016-1758 AMO1) for this study.

### 2.1 Questionnaire design, administration and collection for 2015

A three-page questionnaire on the average period prevalence of, and management practices for, lameness in sheep was developed for the calendar year 2015. The questions were closed and semi-closed and were selected from those used in more detailed questionnaires completed by the same farmers in 2013 (Winter et al., 2015) and 2014 (Grant et al., 2018).

As in the 2013 and 2014 studies, the prevalence of lameness estimated by farmers was the average percentage of ewes lame at any one time over the period, this was validated in King and Green (2011). The time to treatment of lame sheep was the longest time that any sheep was left lame before treatment, categorised into ≤ 3 days (i.e. all sheep treated within 3 days), ≤ 1 week, ≤ 2 weeks and > 2 weeks (i.e. some sheep were not treated within 2 weeks of onset of lameness). Farmers were asked if they practised routine foot trimming, and if they did what percent of feet bled at a routine foot trimming event. This was categorised into 0–2% and ≥ 5%. Farmers were asked if they culled sheep that had been lame, whether they vaccinated ewes against footrot and the number of years they had used the vaccine, and if their sheep mixed with other sheep at planned events e.g. sheep shows or unplanned e.g. insecure boundaries between farms. The questionnaire was internally reviewed by the research team for consistency and ease of completion, it took approximately ten minutes to complete.

In February 2016, 722 farmers were invited by letter to participate in a study to investigate the distribution of serogroups of *D. nodosus* in England, and the practices they used to manage and control lameness in their flocks. These farmers were a subset of the farmers who had completed a questionnaire in 2013 (Winter et al., 2015) and 2014 (Grant et al., 2018) who had indicated that they were willing to participate in future research. A total of 192 (27%) farmers agreed to participate. In February 2016, those farmers were sent the questionnaire together with swabs to sample sheep feet; a reminder was sent to non-respondents after three weeks. In June 2016, a final reminder was sent out with a deadline for responses by the end of July 2016. This deadline was chosen to include farmers who wanted to participate but could not swab sheep feet until July. Questionnaires were returned by 144 / 192 (75%) of the farmers. Responses from a further 18 farmers who participated in the 2013 and 2014 studies (Winter et al., 2015; Grant et al., 2018) who participated in a clinical trial (Witt and Green, 2018) where they completed a more detailed questionnaire which included the same questions, were added to the study. In total completed questionnaires were obtained from 162 farmers. Eight questionnaires that did not include either the year period prevalence of lameness in ewes or the flock size (number of breeding ewes) were excluded from the analysis, which left 154 (95%) usable responses.

### 2.2 Data storage and accuracy

Data from the 2015 questionnaire were entered manually into Microsoft Excel. Data were rechecked once for accuracy against the hardcopies of the questionnaires. Whenever possible, categories within questions with fewer than ten responses were aggregated with the most similar category. The responses from the 2013 and 2014 questionnaires for the farmers that participated in 2015 were retrieved and stored in Microsoft Excel, providing a longitudinal study (Coggon et al., 1997) with data collected retrospectively at three timepoints.

R statistical software version 3.4.2 (R Core Team, 2017) was used for data analysis and modelling.

### 2.3 Representativeness of respondents from invitees

The representativeness of the 154 respondents to the 2015 questionnaire was compared with all 740 (722 plus 18) farmers invited to participate in the 2015 study by geographical location. T-tests (Crawley, 2013) were used to test for a difference in the prevalence of lameness and flock size in 2013 between the 154 respondents and the 740 invitees, and the 154 respondents and the total 1260 respondents to the 2013 questionnaire.

### 2.4 Changes in percentage lameness and managements between 2013, 2014 and 2015

The prevalence of lameness in ewes and the ewe flock size in 2013, 2014 and 2015 were compared using log-transformed data in multilevel models (Dohoo et al., 2003) (Table 1) with no assumed correlation structure using the nlme package (version 3.1-137) (Pinheiro et al., 2018). Year was a fixed effect and flock a random effect in all models. The analysis was conducted with both the 128 flocks who gave data on flock size and prevalence of lameness for all three years, and all 154 respondents to the 2015 questionnaire. Post-hoc analysis was conducted with Tukey HSD (Crawley, 2013) using the multcomp package (Hothorn et al., 2008). For the 154 farmers who answered the 2015 questionnaire, the percentage of farmers that practised managements associated with lameness in 2013 (Winter et al., 2015) were compared with the percentage of farmers who practised those managements in 2014 and 2015 using chi-squared tests and Fisher’s exact tests (Crawley, 2013).

**Table 1:** Multilevel models of the flock size and prevalence of lameness in ewes in all 154 participating English sheep flocks who completed questionnaires on lameness in their sheep in 2013, 2014 and 2015, and the 128 who answered all questions on the flock size and the prevalence of lameness in ewes in each year.

Variable		2013	2014	2015
154 flocks				
Ewe flock size	Median	400	400	400
	IQR	243 – 608	250 – 600	241 – 600
	Range	25 – 5,500	4 – 6000	3 – 6000
	Number of respondents	154	128	154
Prevalence of lameness in ewes	Geometric mean^a^	3.4%	3.1%	4.2%
	95% CI	2.9 – 3.9%	2.8 – 3.6%	3.7 – 4.7%
	Range	0.0 – 40.0%	0.4 – 25.0%	0.5 – 25.0%
	Number of respondents	153	128	154

128 flocks				
Ewe flock size	Median	400	400	400
	IQR	250 – 613	250 – 600	265 – 600
	Range	25 – 5,500	4 – 6000	50 – 6000
Prevalence of lameness in ewes	Geometric mean^b^	3.3%	3.1%	4.1%
	95% CI	2.8 – 3.9%	2.8 – 3.6%	3.6 – 4.6%
	Range	0.0 – 40.0%	0.4 – 25.0%	0.5 – 25.0%

All other pairwise comparisons not significant (p = 0.615 – 0.992).

a Geometric mean prevalence of lameness in ewes from multilevel model significantly different between 2013 and 2015 (p = 0.017), and 2014 and 2015 (p = 0.002).

b Geometric mean prevalence of lameness in ewes from multilevel model significantly different between 2013 and 2015 (p = 0.033), and 2014 and 2015 (p = 0.005).

### 2.5 Identification of management practices associated with flock prevalence of lameness in 2015

The flock prevalence of lameness had an overdispersed distribution, the dispersion parameter of the models (residual deviance divided by the residual degrees of freedom) was greater than one, in both negative binomial models, which assume a negative binomial distribution, and overdispersed Poisson (quasi-Poisson) models, which leave the dispersion parameter unrestricted, which were investigated to identify the model with the best fit (Ver Hoef and Boveng, 2007). Best fit was tested by ranking the predicted number of lame sheep per flock in deciles and comparing with the observed number of lame sheep from each model. A multivariable quasi-Poisson regression model was the best fit and so this was used.

The model took the form:

Observed number of lame ewes*j* ∼ ⍺ + offset + β*j*X*j* + e*j*

Where ∼ is a natural log link, ⍺ is the intercept, the offset is the natural log of the expected number of lame ewes (calculated internally from the flock size), β*j* are coefficients for a vector of X*j* farmer managements which vary by farm *j* and e*j* is the residual random error.

Each variable was tested in a univariable model and the multivariable model was then built using a manual forward stepwise procedure adding the term with the greatest decrease in AIC at each iteration. Once the addition of further variables no longer improved model fit, all the variables were retested in the model to check for residual confounding (Cox and Wermuth, 1996). The model fit was tested by comparing the predicted and observed number of lame sheep per flock ranked in deciles and visually assessed. The model was re-run including only the 128 farmers who provided data on flock size and prevalence of lameness for all three years, and also excluding the 18 farms with data from the clinical trial.

### 2.6 Population attributable fractions of managements associated with the prevalence of lameness

The percentage of the 154 farmers using the management practices (risk factors) in the multivariable model for 2015 were compared with the percentage of the same farmers practising those managements in 2013 and 2014 using chi-squared tests (Crawley, 2013).

In addition, for each risk factor in the multivariable model, the attributable fraction (AF) in the exposed flocks (where the risk factor was present) and the population attributable fraction (PAF) (the proportion of the national lameness attributable to the risk factor) were calculated. Using:

AF = (RR – 1)/RR and PAF = AF (a_1_ / m_1_)

where RR is the risk ratio for a risk factor, a_1_ is the number of flocks exposed to the risk factor and m_1_ is the total number of flocks in the model (Dohoo et al., 2003).

## 3 Results

### 3.1 Representativeness of respondents

**Fig. 1:**
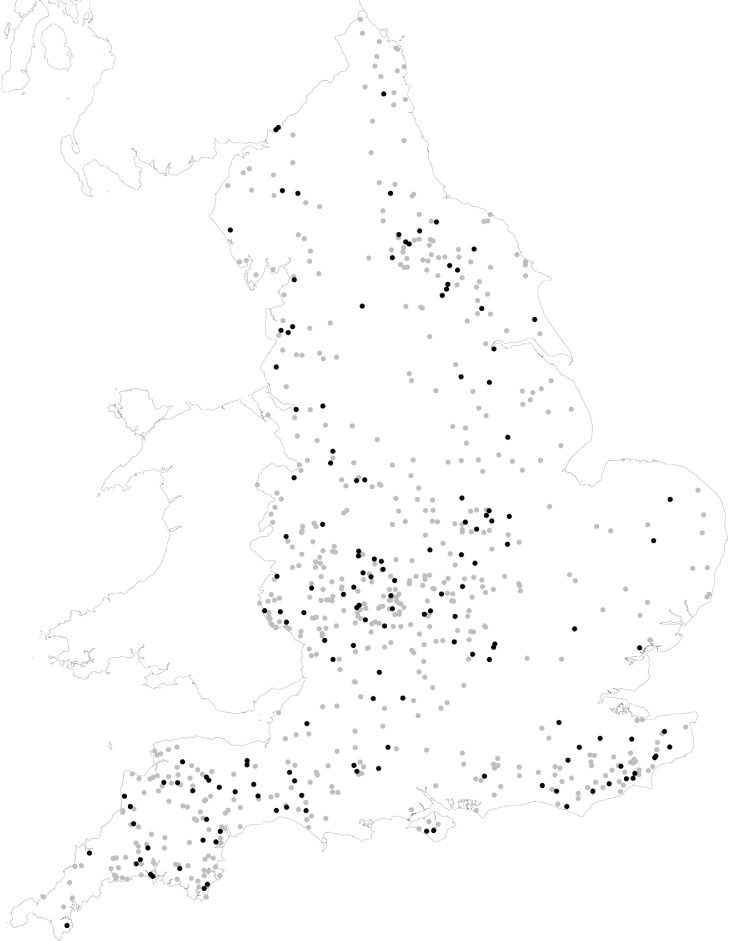
Locations of 722 English sheep farmers who had completed questionnaires on sheep lameness in 2013 or 2014 and were invited to participate in the study (grey), and the 154 farmers who participated by completing a questionnaire regarding lameness in their sheep in 2015 (black).

There was no significant difference in the flock geometric mean period prevalence of lameness in 2013 between the 154 participating and the 740 invited farmers (3.4% and 3.5% respectively) or the total 1260 respondents to the 2013 questionnaire (3.5%) (Winter et al., 2015). There was also no significant difference in flock size between the participating and invited farmers and all respondents of the 2013 questionnaire. There was no difference in geographical location of the 740 farmers invited to participate in the study and the 154 participants (Fig. 1).

### 3.2 Multilevel models of prevalence of lameness in ewes and chi-squared and Fisher’s exact test of managements for lameness 2013–2015

The flock period prevalence of lameness in ewes for the 128 flocks that gave the prevalence of lameness and flock size in all three years was significantly higher in 2015 (4.1%) than in both 2013 (3.3%) and 2014 (3.2%) in the multilevel models (Table 1). This result was the same when all 154 farmers were included in the multilevel models.

There was a large significant reduction in the proportion of farmers that treated all sheep ≤ 3 days of onset of lameness (28.6% in 2015 compared with 50.6% in 2013 and 40.9% in 2014) (Table 2). There was a large, significant reduction in the proportion of farmers who practised routine foot trimming in 2015 (19.5%) compared with 2013 (55.2%) and 2014 (38.3%). The proportion of farmers who carried out routine trimming but did not cause bleeding (7.1%, 11.9%, 6.7% in 2013, 2014, 2015) did not change. Significantly more farmers culled sheep because they had been lame in 2015 than in 2013 and 2014, 81.8% compared with 49.4% and 47.4% respectively. Significantly more farmers used Footvax™, a vaccine against footrot, in 2015 (29.2%) compared with 2013 (14.3%) and 2014 (14.3%). There were no other significant changes in management between 2013 or 2014 and 2015 in this relatively small sample of 154 flocks, and no significant change in any management between 2013 and 2014.

**Table 2:** Number and percentage of 154 English sheep flocks by management practices associated with lameness from questionnaires completed in 2013, 2014 and 2015.

Variable	2013		2014		2015		
	N	%	N	%	N	%	
Lowest locomotion score at which the farmer recognised sheep as lame (Kaler et al., 2009)	1	80	51.9	72	46.8	Not investigated	
	2	55	35.7	48	31.2		
	≥3	18	11.7	10	6.5		

Number of lame sheep at locomotion score when farmers treated them	1	19	12.3	25	16.2	Not investigated	
	2 – 5	77	50.0	65	42.2		
	6 – 10	31	20.1	25	16.2		
	>10	25	16.2	13	8.4		
	Did not treat individuals	0	0.0	1	0.6		

**Time to treatment of all lame sheep**	**≤3 days**	**78**	**50.6**	**63**	**40.9**	**44**	**28.6**
	**≤1 week**	**55**	**35.7**	**51**	**33.1**	**74**	**48.1**
	**≤2 weeks**	**15**	**9.7**	**14**	**9.1**	**24**	**15.6**
	**>2 weeks**	**3**	**1.9**	**2**	**1.3**	**10**	**6.5**

Ease of catching individual lame sheep	Easy/very easy	20	13.0	19	12.3	Not investigated	
	Neither easy or difficult	67	43.5	54	35.1		
	Difficult/very difficult	65	42.2	56	36.4		

Method of catching individual sheep: corner of field	No	99	64.3	Not investigated		Not investigated	
	Yes	55	35.7				
Method of catching lame sheep: dog that can catch individuals	No	132	85.7	Not investigated		Not investigated	
	Yes	22	14.3				
**Proportion of sheep that bled during a routine foot trim, per year**	**Did not trim**	**63**	**40.9**	**72**	**46.8**	**122**	**79.2**
	**Zero**	**6**	**3.9**	**7**	**4.5**	**2**	**1.3**
	**<1%**	**6**	**3.9**	**6**	**3.9**	**2**	**1.3**
	**1 – 2%**	**35**	**22.7**	**29**	**18.8**	**16**	**10.4**
	**>2 – < 5%**	**11**	**7.1**	**1**	**0.6**	**0**	**0.0**
	**5 – <10%**	**18**	**11.7**	**11**	**7.1**	**7**	**4.5**
	**≥10%**	**9**	**5.8**	**5**	**3.2**	**3**	**1.9**

Footbath all ewes ever over the past year	No	52	33.8	50	32.5	64	41.6
	Yes	102	66.2	81	52.6	90	58.4

Footbath to treat footrot	No	98	63.6	Not investigated		Not investigated	
	Yes	56	36.4				

Footbath to prevent ID	No	96	62.3	Not investigated		Not investigated	
	Yes	58	37.7				

Occasion footbathed: at turnout	No	150	97.4	127	82.5	Not investigated	
	Yes	4	2.6	4	2.6		

Occasion footbathed: new sheep on arrival	No	101	65.6	78	50.6	Not investigated	
	Yes	30	19.5	30	19.5		
	No new sheep	22	14.3	21	13.6		

**Culled sheep previously lame**	**No**	**71**	**46.1**	**56**	**36.4**	**27**	**17.5**
	**Yes**	**76**	**49.4**	**73**	**47.4**	**126**	**81.8**

Relied on memory to identify culls	No	151	98.1	Not investigated		Not investigated	
	Yes	3	1.9				

Avoided selling ewes for breeding from repeatedly lame mothers	No	149	96.8	Not investigated		Not investigated	
	Yes	5	3.2				

**Vaccinated ewes with Footvax^TM^**	**No**	**132**	**85.7**	**109**	**70.8**	**109**	**70.8**
	**Yes**	**22**	**14.3**	**22**	**14.3**	**45**	**29.2**

Length of time vaccinating against footrot	>5 years	Not investigated		Not investigated		15	9.7
	>2 – 5 years					12	7.8
	>1 – 2 years					12	7.8
	>0 – 1 year					12	7.8
	Did not vaccinate					96	62.3

Checked feet of new sheep on arrival	Never	16	10.4	Not investigated		Not investigated	
	Sometimes	18	11.7				
	Usually	36	23.4				
	Always	58	37.7				
	No new arrivals	23	14.9				

Isolated new sheep on arrival	Did not isolate	10	6.5	5	3.2	Not investigated	
	Isolated for < 3 weeks	75	48.7	60	39.0		
	Isolated for ≥ 3 weeks	44	28.6	41	26.6		
	No new arrivals	23	14.9	23	14.9	23	14.9

Sheep mixed with other flocks	No	131	85.1	Not investigated		144	93.5
	Yes	17	11.0			10	6.5
	Do not know	2	1.3			0	0.0

Sheep left farm then returned: for shows	No	148	96.1	Not investigated		148	96.1
	Yes	6	3.9			6	3.9

Sheep left farm then returned: for summer grazing	No	126	81.8	Not investigated		Not investigated	
	Yes	28	18.2				

Sheep left farm then returned: for market	No	149	96.8	Not investigated		Not investigated	
	Yes	5	3.2				

Farm location	Upland	12	7.8	Not investigated		Not investigated	
	Hill	2	1.3				
	Lowland	138	89.6				

Organic status	Not organic	143	92.9	Not investigated		Not investigated	
	Organic	9	5.8				

Production of breeding stock	No	119	77.3	Not investigated		Not investigated	
	Yes	35	22.7				

BOLD: significant differences in farmer practices in 2015 compared with both 2013 and 2014 (Wald’s test p < 0.05). N: number of farmers; %: percent of farmers. There were no significant differences in farmer practices between 2013 and 2014. “No response” was a category in each variable, results not shown.

### 3.3 Multivariable quasi-Poisson regression model of risk factors for lameness in sheep

The univariable model results are in Supplementary Table 1. The dispersion parameter of the final model was 8.8. In the multivariable model, four variables were significantly associated with the prevalence of lameness in ewes in 2015 (Table 3). The prevalence of lameness was higher in flocks where farmers treated all lame sheep ≤ 1 week (RR 1.57, 95% CI: 1.18–2.13), ≤ 2 weeks (RR 1.49, 95% CI: 1.02–2.18) or > 2 weeks (RR 1.73, 95% CI: 1.10–2.65) of onset of lameness, compared with flocks where farmers treated all sheep ≤ 3 days of onset of lameness. The prevalence of lameness was greater in flocks where ≥ 5% of sheep feet bled during routine foot trimming (RR 1.79, 95% CI: 1.26–2.48) than in flocks where routine foot trimming was not practised. The prevalence of lameness was higher in flocks where annual vaccination against footrot had been practised for between 2 and 5 years (RR 2.05, 95% CI: 1.31–3.24) and ≤ 1 year (RR 2.83, 95% CI: 1.72–4.66), and when vaccination against footrot was not practised (RR 1.70, 95% CI: 1.20–2.48) compared with flocks that had been vaccinated annually for > 5 years. Flocks that mixed with other flocks (planned or accidental) had a higher prevalence of lameness (RR 1.58, 95% CI: 1.06–2.27) than those where sheep did not mix with other flocks. The model fit was visually good (Supplementary Fig. 1). There was no change to the model results when only the 128 farmers who provided lameness data for all three years of the study were included or when the 18 farmers in the clinical trial were excluded (data not shown). The management practices significantly different between 2013 and 2015 were also significantly different between 2014 and 2015 (data not shown).

**Table 3:** Multivariable quasi-Poisson regression model of risk factors associated with the period prevalence of lameness in ewes in 154 English sheep flocks in 2015.

Variable	Number	Percent	Risk Ratio	95% CI	
Time to treatment of all lame sheep					
≤3 days	44	28.6	1.00		
≤1 week	74	48.1	**1.57**	**1.18**	**2.13**
≤2 weeks	24	15.6	**1.49**	**1.02**	**2.18**
>2 weeks	10	6.5	**1.73**	**1.10**	**2.65**

Percent of sheep that bled during routine foot trimming					
No routine foot trimming	122	79.2	1.00		
0–2%	20	13.0	1.26	0.91	1.73
≥5%	10	6.5	**1.79**	**1.26**	**2.48**

Length of time vaccinating against footrot					
>5 years	15	9.7	1.00		
>2–5 years	12	7.8	**2.05**	**1.31**	**3.24**
>1–2 years	12	7.8	1.11	0.67	1.84
>0–1 year	12	7.8	**2.83**	**1.72**	**4.66**
Did not vaccinate	96	62.3	**1.70**	**1.20**	**2.48**
Sheep mixed with other flocks					
No	144	93.5	1.00		
Yes	10	6.5	**1.58**	**1.06**	**2.27**

BOLD: categories significantly different from the baseline (Wald’s test p < 0.05). CI: confidence intervals. Model coefficient: -0.957, Standard Error: 0.189. “No response” was a category in each variable, results not shown.

### 3.4 Population attributable fractions (PAF) of risk factors for lameness in ewes and farmer changes in management practices between 2013 and 2015

Up to 65.3% of the prevalence of lameness was explained by the model (Table 4). The percentage of farmers that treated all lame sheep promptly, that is, ≤ 3 days of onset of lameness, fell by 22% from 50.6% to 28.6% (Table 2) between 2013 and 2015 and the PAF of lameness attributable to not treating all sheep ≤ 3 days of onset of lameness rose from 13.3% in 2013 (Grant et al., 2018) to 25.3% in 2015 (Table 4). Significantly fewer farmers practised routine foot trimming in 2015 compared with 2013, 19.5% and 55.2% respectively, and the proportion of flocks where ≥ 5% feet bled fell by 11% from 17.5% to 6.5%. The PAF of lameness attributable to feet bleeding during routine foot trimming fell from 9.5% to 2.9%. More farmers used Footvax™ in 2015 than in 2013; 29.2% compared with 14.3% respectively. In 2015, vaccinating against footrot annually for < 6 years had the largest PAF of 34.7%. The number of years a flock had been vaccinated against footrot was not investigated in 2013 and so a change in this specific vaccination behaviour could not be investigated (Table 2), however the PAF for not vaccinating ewes at all in 2013 compared with vaccinating once per year (regardless of duration of vaccination) was 3.3%. There was no significant change in the percentage of farmers whose sheep did not mix with other flocks (85.1% in 2013 and 93.5% in 2015) and the PAF for this practice was 2.4% in 2015. Attending sheep shows had a PAF of 1.3% in 2013.

**Table 4:** Attributable fractions and population attributable fractions of four management practices associated with the prevalence of lameness in ewes in 154 English sheep flocks in 2015.

Variable	Farmers (%)	RR	AF (%)	PAF (%)
Time to treatment of all lame sheep: ≤3 days	28.6	1.00	0.0	0.0
Time to treatment of all lame sheep: ≤1 week	48.1	1.57	36.4	17.5
Time to treatment of all lame sheep: ≤2 weeks	15.6	1.49	32.8	5.1
Time to treatment of all lame sheep: >2 weeks	6.5	1.73	42.1	2.7

No routine foot trimming	79.2	1.00	0.0	0.0
≥5% sheep bled during routine foot trimming	6.5	1.79	44.1	2.9

Vaccinating >5 years	9.7	1.00	0.0	0.0
Did not vaccinate	62.3	1.70	41.3	25.7
Vaccinating ≤1 year	7.8	2.83	64.7	5.0
Vaccinating >2–≤5 years	7.8	2.05	51.3	4.0

Sheep not mixed with other flocks	93.5	1.00	0.0	0.0
Sheep mixed with other flocks	6.5	1.58	36.7	2.4

RR: Risk ratio; AF: Attributable fraction (exposed); PAF: Population attributable fraction.

## 4 Discussion

This paper provides highly novel evidence from a three-year longitudinal study of a cohort of English sheep farmers that when farmers stop following a robust evidence-based management practice to minimise the prevalence of lameness in sheep, that is individual treatment of all sheep in ≤ 3 days of onset of lameness, the prevalence of lameness increases. In veterinary research there are few examples of diseases with robust evidence-based management practices, where new management practices have been adopted and a reduction in prevalence of disease was observed, as there is for lameness in sheep in England (Winter et al., 2015; Grant et al., 2018). We believe the current study is the first example to illustrate that stopping a beneficial behaviour leads to an increase in prevalence of a disease in a cohort of commercial farms. The evidence from our research strengthens the evidence that treatment in ≤ 3 days of onset of lameness is likely to be causal rather than simply an association. Using Bradford Hill’s (1965) criteria for causality our evidence provides temporality and reversibility, the latter that removal of an exposure leads to a change in disease occurrence: in our study, removal of prompt treatment of lame sheep increased the flock prevalence of lameness.

The geometric mean flock prevalence of lameness in ewes was 24% and 32% higher in 2015 than in 2013 and 2014 respectively. The key detrimental change in management of lameness was that the percentage of farmers who treated lame sheep in ≤ 3 days fell by 44% between 2013 (Winter et al., 2015) and 2015, and the PAF attributable to this change rose from 13.3% in 2013 (Grant et al., 2018) to 25.3% in 2015 (Table 4). The management changes farmers made with vaccination, culling and foot trimming align with previous evidence for management of lameness (Winter et al., 2015), however, the increase in the proportion of farmers using these practices did not offset the effect from the reduction in prompt treatment of lame sheep in 2015, and so the overall effect was an increase in the period prevalence of lameness.

The proportion of farmers that practised routine foot trimming has reduced substantially since 2004 (Winter et al., 2015; Grant et al., 2018) and the reduction in the current study from 55.2% in 2013 and 38.3% in 2014 to 19.4% in 2015 is remarkable. Winter et al. (2015) identified that excessive trimming into sensitive tissue, causing bleeding, was the risk associated with foot trimming that increased the prevalence of lameness. The proportion of flocks where excessive trimming occurred fell by 65% between 2013 and 2015, and consequently the PAF from routine foot trimming was < 3% in the current study. If generalisable, this is an excellent result for sheep welfare. It is notable that the percentage of feet that bled in flocks where routine foot trimming was conducted did not change, therefore farmers are not improving their ability to trim feet and not cause bleeding. We conclude that the recommendation to stop foot trimming is still the most effective approach to avoid over trimming and to save considerable farmer time (Wassink et al., 2010a).

The proportion of flocks that were vaccinated against footrot has increased since 2013 (Winter et al., 2015). This is the first study to find an association with the duration of the use of Footvax™ and the prevalence of lameness. The pattern of association was complex with a protective effect of annual vaccination of flocks for > 5 years but a higher prevalence of lameness in flocks vaccinated for ≤ 5 years compared with flocks not vaccinated (Table 3). It is not clear why this pattern of risk was observed. One explanation is that farmers who have used the vaccine for > 5 years continue to use it because it has been effective, another is that it takes considerable time for the vaccine to improve control of lameness. It has been estimated that Footvax™ reduces the prevalence of footrot by 20%–70% (Hindmarsh et al., 1989; Duncan et al., 2012; Winter et al., 2015). There were relatively few farmers vaccinating in the current study and further study is required to understand the impact of long-term vaccination against footrot.

Mixing a flock with other flocks was associated with a small PAF because it was a rare event. Mixing included both planned mixing, e.g. shows, and unplanned, e.g. mixing of neighbouring flocks due to poor fencing. Mixing has been associated with increased prevalence of ID (Wassink et al., 2004) and lameness (Winter et al., 2015), and this is likely to be due to introduction of strains of footrot and CODD (Angell et al., 2014; Dickins et al., 2016), both infectious causes of lameness.

One question from the current study is why have so many farmers stopped treating lame sheep within three days of onset of lameness. Data were collected for 2013, 2014 and 2015. There was a small but non-significant change in management practices between 2013 and 2014 and a small non-significant difference in prevalence of lameness (Table 1, Table 2). The big change in prevalence of lameness and management practices was observed in 2015. From 2014, a five-point lameness control plan (EBLEX, 2014) was promoted widely throughout England and used by the manufacturers of the footrot vaccine, Footvax™ (MSD Animal Health, 2014). The ‘Five Point Plan’ includes management of quarantine, treatment, vaccination, culling and selection of replacement stock. A case-study of three flocks using the approach reported that the flock prevalence of lameness decreased from 7.4% to < 2% within three years in one flock, and from an unknown prevalence to < 3% in two flocks (Clements and Stoye, 2014). The promotion of the ‘Five Point Plan’ is temporally associated with the change in management practices among the 154 farmers in the current study. These farmers’ behaviour in 2013 and 2014 was similar to all respondents in 2013 (Winter et al., 2015) and 2014 (Grant et al., 2018) and so it is likely that the changes in practices observed have occurred in sheep flocks across England. One explanation for the change in behaviours and consequent increase in prevalence of lameness is that although the ‘Five Point Plan’ recommends both individual and flock managements, farmers have been more receptive to adopting the whole flock managements of vaccination, culling and avoiding foot trimming, and they have reduced activity on individual treatment of lame sheep in ≤ 3 days of onset of lameness (Wassink et al., 2010a; Winter et al., 2015). Wassink et al. (2010a), proposed that because farmers preferred flock managements, they would adopt these in preference to individual treatment, although they know the latter is more effective, and that knowledge exchange providers needed to be aware of this when promoting managements for lameness. Whilst this detrimental change in behaviour is unfortunate, it could be reversed, and if all farmers used prompt treatment the geometric mean flock prevalence of lameness would fall to 3.1% (Table 4). This reduction in lameness could be achieved within months (Wassink et al., 2010b). Research to identify barriers to, and methods for, catching individual lame sheep is important to help some farmers take up this practice. In contrast, the flock prevalence of lameness would fall to 2.7% in a minimum of six years if all flocks were vaccinated annually against footrot for > 5 years, assuming the benefit of vaccination in all flocks is generalisable, (the number of farmers using vaccine in the current study was small). Further reduction in foot trimming (Winter et al., 2015; current paper) and improved quarantine and biosecurity (Winter et al., 2015; Witt and Green, 2018) would also reduce the prevalence of lameness, possibly to ≤2%, the FAWC target for 2021 (FAWC, 2011).

## 5 Conclusions

We provide robust evidence from a cohort of 154 farmers in a three-year longitudinal study that when farmers stop implementing an evidence-based behaviour, prompt treatment of lame sheep, there is a consequent increase in flock period prevalence of lameness. The change in behaviour might be explained by cognitive dissonance, because farmers prefer flock-based activities of stopping foot trimming and using vaccination over less preferred, but more effective, activities of prompt individual treatment of lame sheep. We provide new evidence that annual vaccination against footrot for > 5 years was associated with reduced prevalence of lameness which, if causal and generalisable, and combined with individual treatment of lame sheep within 3 days of onset of lameness, would provide good control of lameness and reduce the prevalence of lameness in sheep to ≤ 2%, the FAWC (2011) target for 2021, and a goal that is important for health, welfare and productivity in the English sheep industry.

## Declaration of Competing Interest

None.
