# Supplementary material for: Increase in the flock prevalence of lameness in ewes is associated with a reduction in farmers using evidence-based management of prompt treatment: A longitudinal observational study of 154 English sheep flocks 2013–2015
Source: Prev Vet Med. 2019 Dec 1;173:104801. doi: 10.1016/j.prevetmed.2019.104801 (PMC6899501; doi:10.1016/j.prevetmed.2019.104801)
Supplement: Supplementary file 1 [file mmc1.docx]

**Increase in the flock prevalence of lameness in ewes is associated with a reduction in farmers using evidence-based management of prompt treatment: a longitudinal observational study of 154 English sheep flocks 2013 – 2015 – supplementary data**

**Supplementary Table 1**

The univariable model results of variables tested in the multivariable quasi-Poisson regression model for lameness in ewes in 154 English sheep flocks.

| **Variable** | **Farmers** | | **RR** | **95% CI** | |
| --- | --- | --- | --- | --- | --- |
|  | **N** | **%** |  |  |  |
| Used foot trimming to treat SFR in ewes | | | | | |
| No | 62 | 40.3 | 1.00 |  |  |
| Yes | 89 | 57.8 | 1.10 | 0.86 | 1.42 |
| Used antibiotic injection to treat SFR in ewes | | | | | |
| No | 17 | 11.0 | 1.00 |  |  |
| Yes | 134 | 87.0 | 0.70 | 0.46 | 1.12 |
| Used foot spray to treat SFR in ewes | | | | | |
| No | 7 | 4.5 | 1.00 |  |  |
| Yes | 144 | 93.5 | 0.96 | 0.56 | 1.85 |
| Separated lame individuals to treat SFR in ewes | | | | | |
| No | 103 | 66.9 | 1.00 |  |  |
| Yes | 48 | 31.2 | 0.93 | 0.72 | 1.19 |
| Used another treatment to treat SFR in ewes | | | | | |
| No | 150 | 97.4 | 1.00 |  |  |
| Yes | 1 | 0.6 | 0.82 | 0.08 | 3.06 |
| Used foot trimming to treat SFR in lambs | | | | | |
| No | 109 | 70.8 | 1.00 |  |  |
| **Yes** | **41** | **26.6** | **1.34** | **1.02** | **1.74** |
| Used antibiotic injection to treat SFR in lambs | | | | | |
| No | 63 | 40.9 | 1.00 |  |  |
| Yes | 87 | 56.5 | 1.13 | 0.87 | 1.47 |
| Used foot spray to treat SFR in lambs | | | | | |
| No | 20 | 13.0 | 1.00 |  |  |
| **Yes** | **130** | **84.4** | **1.85** | **1.23** | **2.93** |
| Separated lame individuals to treat SFR in lambs | | | | | |
| No | 126 | 81.8 | 1.00 |  |  |
| **Yes** | **24** | **15.6** | **1.43** | **1.00** | **1.98** |
| Used another treatment to treat SFR in lambs | | | | | |
| No | 149 | 96.8 | 1.00 |  |  |
| Yes | 1 | 0.6 | 0.82 | 0.08 | 3.07 |
| Time to treatment of all lame sheep | | | | | |
| ≤ 3 days | 44 | 28.6 | 1.00 |  |  |
| **≤ 1 week** | **74** | **48.1** | **1.85** | **1.37** | **2.54** |
| **≤ 2 weeks** | **24** | **15.6** | **1.75** | **1.17** | **2.60** |
| **> 2 weeks** | **10** | **6.5** | **1.87** | **1.14** | **2.99** |
| Used routine foot trimming to manage SFR | | | | | |
| No | 126 | 81.8 | 1.00 |  |  |
| **Yes** | **28** | **18.2** | **1.37** | **1.01** | **1.81** |
| Used routine foot bathing to manage SFR | | | | | |
| No | 64 | 41.6 | 1.00 |  |  |
| Yes | 90 | 58.4 | 1.14 | 0.89 | 1.49 |
| Used footrot vaccination to manage SFR | | | | | |
| No | 96 | 62.3 | 1.00 |  |  |
| Yes | 58 | 37.7 | 0.92 | 0.72 | 1.18 |
| Separated lame sheep to manage SFR | | | | | |
| No | 96 | 62.3 | 1.00 |  |  |
| Yes | 58 | 37.7 | 1.04 | 0.82 | 1.33 |
| Used another management to manage SFR | | | | | |
| No | 151 | 98.1 | 1.00 |  |  |
| Yes | 3 | 1.9 | 0.86 | 0.19 | 2.39 |
| Percentage of sheep that bled during routine foot trimming | | | | | |
| No routine foot trimming | 122 | 79.2 | 1.00 |  |  |
| 0 – 2% | 20 | 13.0 | 1.17 | 0.80 | 1.65 |
| **≥ 5%** | **10** | **6.5** | **1.94** | **1.30** | **2.79** |
| Sheep arriving on farm quarantined for at least 3 weeks | | | | | |
| N/A | 21 | 13.6 | 1.00 |  |  |
| Always | 76 | 49.4 | 1.08 | 0.73 | 1.66 |
| Sometimes | 31 | 20.1 | 1.14 | 0.72 | 1.83 |
| Never | 23 | 14.9 | 1.34 | 0.83 | 2.19 |
| Sheep that had been lame were culled | | | | | |
| After 1 or 2 bouts | 28 | 18.2 | 1.00 |  |  |
| After 3 or more bouts | 10 | 6.5 | 1.34 | 0.72 | 2.38 |
| When persistently lame / severe disease / misshapen hoof | 88 | 57.1 | 1.21 | 0.86 | 1.75 |
| Never | 27 | 17.5 | 1.24 | 0.78 | 1.98 |
| Vaccinated with Footvax^TM^ | | | | | |
| Some sheep, but not all ewes | 13 | 8.4 | 1.00 |  |  |
| No sheep | 96 | 62.3 | 1.36 | 0.95 | 2.00 |
| Ewes | 45 | 29.2 | 1.40 | 0.95 | 2.10 |
| Frequency of Footvax^TM^ use | | | | | |
| Never | 96 | 62.3 | 1.00 |  |  |
| Once/year | 47 | 30.5 | 1.08 | 0.83 | 1.39 |
| **> once/year** | **11** | **7.1** | **0.60** | **0.39** | **0.89** |
| Length of time vaccinating against footrot | | | | | |
| > 5 years | 15 | 9.7 | 1.00 |  |  |
| **Did not vaccinate** | **96** | **62.3** | **1.92** | **1.33** | **2.85** |
| **> 0 – 1 year** | **12** | **7.8** | **3.38** | **2.01** | **5.64** |
| > 1 – 2 years | 12 | 7.8 | 1.36 | 0.80 | 2.28 |
| **> 2 – 5 years** | **12** | **7.8** | **2.29** | **1.44** | **3.67** |
| Sheep purchased | | | | | |
| Yes | 131 | 85.1 | 1.00 |  |  |
| No | 23 | 14.9 | 1.06 | 0.73 | 1.49 |
| Sheep purchased from market | | | | | |
| No | 65 | 42.2 | 1.00 |  |  |
| **Yes** | **87** | **56.5** | **1.39** | **1.09** | **1.78** |
| Sheep purchased from private farm sale | | | | | |
| No | 86 | 55.8 | 1.00 |  |  |
| **Yes** | **66** | **42.9** | **0.73** | **0.57** | **0.93** |
| Regions sheep were purchased from | | | | | |
| The South East & East Anglia | 21 | 13.6 | 1.00 |  |  |
| No purchase made | 23 | 14.9 | 1.41 | 0.87 | 2.29 |
| **The North & Scotland** | **32** | **20.8** | **1.56** | **1.05** | **2.38** |
| The Midlands & Wales | 31 | 20.1 | 1.37 | 0.87 | 2.19 |
| The South West | 26 | 16.9 | 1.58 | 0.98 | 2.56 |
| Don’t know | 1 | 0.6 | 1.40 | 0.08 | 6.47 |
| Multiple regions | 17 | 11.0 | 1.07 | 0.64 | 1.77 |
| Frequency of sheep purchases over last 5 years | | | | | |
| > once/year | 45 | 29.2 | 1.00 |  |  |
| ≤ once/year | 83 | 53.9 | 1.01 | 0.77 | 1.32 |
| Prevalence of lameness in lambs in the flock | | | | | |
| **For each percent increase in prevalence of lamb lameness** | **154** | **100.0** | **1.05** | **1.03** | **1.06** |
| Ewe stocking rate | | | | | |
| < 4 /acre | 69 | 44.8 | 1.00 |  |  |
| ≥ 4 /acre | 82 | 53.2 | 1.12 | 0.87 | 1.43 |
| Sheep mixed with other flocks | | | | | |
| No | 144 | 93.5 | 1.00 |  |  |
| Yes | 10 | 6.5 | 1.43 | 0.90 | 2.16 |
| Flock shared grazing with cattle | | | | | |
| Yes | 72 | 46.8 | 1.00 |  |  |
| No | 82 | 53.2 | 0.97 | 0.76 | 1.24 |
| Avoided selecting replacement ewes from repeatedly lame mothers | | | | | |
| Yes | 66 | 42.9 | 1.00 |  |  |
| No | 87 | 56.5 | 1.03 | 0.80 | 1.33 |
| BOLD: Significant associations with lameness in ewes (Wald’s test p<0.05). N: number of farmers; RR: risk ratio; CI: confidence intervals; SFR: severe footrot. | | | | | |

**Supplementary Figure 1**

The predicted and observed deciles of the number of lame sheep per flock from a multivariable quasi-Poisson regression model with four explanatory variables for 154 English sheep flocks, ranked by the observed data.
